# Open Genes—a new comprehensive database of human genes associated with aging and longevity

**DOI:** 10.1093/nar/gkad712

**Published:** 2023-09-04

**Authors:** Ekaterina Rafikova, Nikolay Nemirovich-Danchenko, Anna Ogmen, Anna Parfenenkova, Anastasiia Velikanova, Stanislav Tikhonov, Leonid Peshkin, Konstantin Rafikov, Olga Spiridonova, Yulia Belova, Timofey Glinin, Anastasia Egorova, Mikhail Batin

**Affiliations:** Open Longevity, 15260 Ventura Blvd, STE 2230, Sherman Oaks, CA 91403, USA; Open Longevity, 15260 Ventura Blvd, STE 2230, Sherman Oaks, CA 91403, USA; Faculty of Cytology and Genetics, National Research Tomsk State University, Tomsk 634050, Russia; Open Longevity, 15260 Ventura Blvd, STE 2230, Sherman Oaks, CA 91403, USA; Department of Molecular Biology and Genetics, Bogazici University, Istanbul 34342, Turkey; Open Longevity, 15260 Ventura Blvd, STE 2230, Sherman Oaks, CA 91403, USA; Open Longevity, 15260 Ventura Blvd, STE 2230, Sherman Oaks, CA 91403, USA; Faculty of Bioengineering and Bioinformatics, Moscow State University, Moscow 119991, Russia; Department of Systems Biology, Harvard Medical School, Boston, MA 02115, USA; Open Longevity, 15260 Ventura Blvd, STE 2230, Sherman Oaks, CA 91403, USA; Open Longevity, 15260 Ventura Blvd, STE 2230, Sherman Oaks, CA 91403, USA; Open Longevity, 15260 Ventura Blvd, STE 2230, Sherman Oaks, CA 91403, USA; Open Longevity, 15260 Ventura Blvd, STE 2230, Sherman Oaks, CA 91403, USA; Endocrine Neoplasia Laboratory, Department of Surgery, University of California, San Francisco, San Francisco, CA 94143, USA; Department of Genetics & Biotechnology, Saint Petersburg State University, Saint Petersburg 199034, Russia; Open Longevity, 15260 Ventura Blvd, STE 2230, Sherman Oaks, CA 91403, USA; Open Longevity, 15260 Ventura Blvd, STE 2230, Sherman Oaks, CA 91403, USA

## Abstract

The Open Genes database was created to enhance and simplify the search for potential aging therapy targets. We collected data on 2402 genes associated with aging and developed convenient tools for searching and comparing gene features. A comprehensive description of genes has been provided, including lifespan-extending interventions, age-related changes, longevity associations, gene evolution, associations with diseases and hallmarks of aging, and functions of gene products. For each experiment, we presented the necessary structured data for evaluating the experiment's quality and interpreting the study's findings. Our goal was to stay objective and precise while connecting a particular gene to human aging. We distinguished six types of studies and 12 criteria for adding genes to our database. Genes were classified according to the confidence level of the link between the gene and aging. All the data collected in a database are provided both by an API and a user interface. The database is publicly available on a website at https://open-genes.org/.

## Graphical Abstract

**Graphical Abstract:**
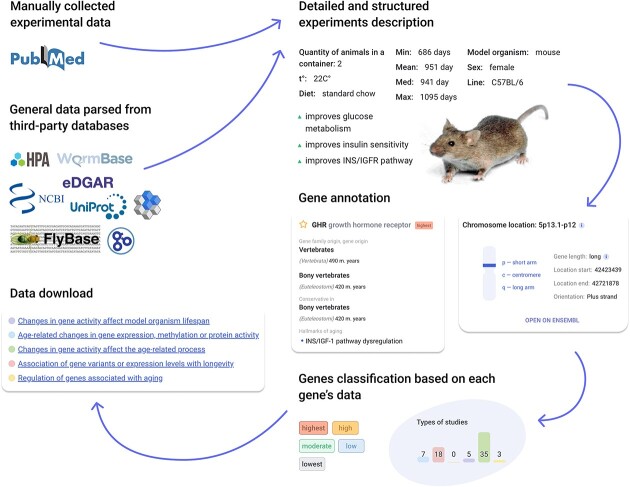

## Introduction

In recent decades, an enormous number of studies on the genetics of aging have been conducted and published. PubMed provides >10 000 papers, published between 2000 and 2021 for an ‘aging genetics’ search request. Several databases were developed in an attempt to structure these studies. Some databases are focused on a specific subject, such as the Human Cellular Senescence Gene Database (HCSGD), which focuses on senescence (1), and SynergyAge, which focuses on the effect of combinations of genetic interventions on the lifespan of model organisms (2). Human Ageing Genomic Resources (HAGR) is a collection of resources about various data on the aging of humans and animals, including age-related alterations, the impact of genetic interventions on the lifespan of model organisms, longevity-associated allelic variants, and others (3–6).

Existing databases are limited to a particular aspect of aging or a specific type of studies, which makes it impossible to compare the results of various experiments and take into account all known interconnections between a given gene and the aging process. In some cases the lack of study details makes it difficult to interpret the experiment's result.

There are five differences between Open Genes and other existing databases on the association between genes and aging:

- Instead of providing the results of different types of experiments in different sections of the database, we have combined all the data in one location and linked to the human genes. This approach, as well as filters by selection criteria and confidence levels on the database website, allows users to sort genes by a variety of parameters and generate lists of genes of interest for further analysis. In addition, all the data about each gene is presented on a separate gene page.
- We provide detailed, structured data for each experiment linking the gene and aging. For example, we specify up to 35 parameters for each lifespan experiment, including the tissue specificity of an intervention, the age of the model organism at the beginning and end of treatment, and the absolute values of the lifespans of control and experimental groups. The details depend on the study design and data availability. This approach allows users to compare experiments and interpret the results more accurately. All datasets are available for download on the Open Genes website.
- We provide manually collected data on gene evolution. Based on phylogenetic studies, the gene origin and the origin of the gene family have been specified, along with a description of the main events in gene evolution for 455 aging-related genes.
- In order to remain objective and precise while linking a particular gene to human aging, and maintain the transparency of the gene selection criteria, we distinguished 6 types of studies and 12 criteria for adding genes to the Open Genes database. The genes were divided into groups with different confidence levels based on the type of data supporting the link between the gene and aging.
- Open Genes has been developed using a modern technology stack, and all code and data have been made publicly available.

The Open Genes database aims to structure and combine all available data on the genetics of aging within a single resource and provide convenient tools for searching, assorting, and comparing genes according to a variety of features: age-related alterations, associations with longevity, lifespan-extending genetical interventions, gene evolution, associations with diseases and hallmarks of aging, interaction with other genes and functions of gene products. Our approach is expected to facilitate the target discovery and significantly reduce the time required to search for genes that are reliably associated with aging.

Open Genes embeds detailed and structured descriptions of experimental setups from over 1700 publications allowing for adequate evaluation of each gene's association with longevity or aging processes. It also aims to assist with the selection of future research directions.

With Open Genes, we identified 25 genes associated with human longevity, whose modifications impact the lifespan of mammals and analyzed the consistency between the change in gene activity that extended the lifespan of mammals and the effect of longevity-associated polymorphisms on the level of gene expression or protein activity. In addition, we identified groups of genes with the consistent and controversial results of life-extending experiments depending on the model organism.

## Results

**Figure 1.**
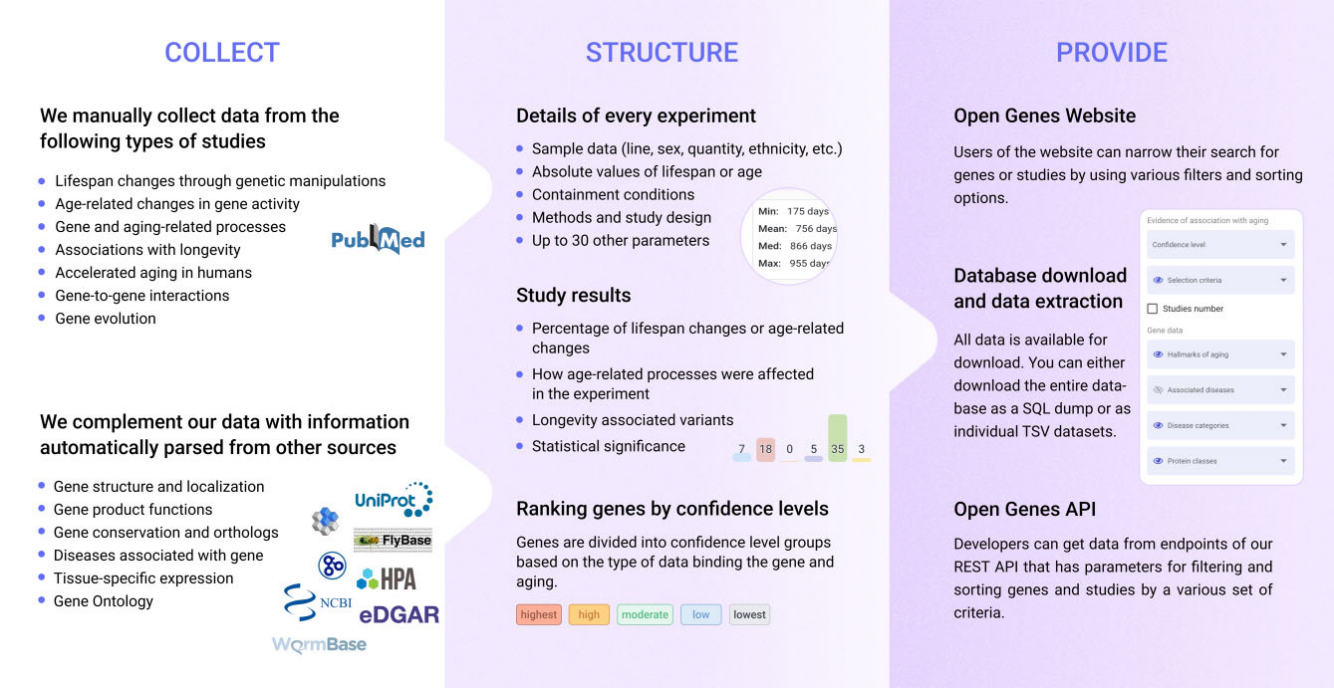
The overview of Open Genes.

Open Genes is a resource that brings together a vast amount of manually collected data on experimental evidence for the gene-aging association and general information on genes, their function, and their association with disease parsed from other databases. The overview of Open Genes is presented in Figure 1. The following sections provide a detailed characterization of the data manually collected from the literature and obtained from third party biological databases.

### Genes selection and classification

#### Genes selection criteria

The Open Genes database aims to collect all available data on the genetics of aging and structure it for analysis and searching for aging therapy targets. All data on experimental evidence of the link between genes and aging are collected manually from the literature.

We collected several types of data regarding each gene. We distinguished 6 types of studies and 12 criteria for adding genes to the Open Genes database:

- Changes in gene activity affect the model organism's lifespan:
  - Changes in gene activity extend the mammalian lifespan
  - Changes in gene activity extend the non-mammalian lifespan
  - Changes in gene activity reduce the mammalian lifespan
  - Changes in gene activity reduce the non-mammalian lifespan
- Age-related changes in gene expression, methylation or protein activity:
  - Age-related changes in humans
  - Age-related changes in mammals
  - Age-related changes in non-mammals
- Changes in gene activity affect the age-related process:
  - Changes in gene activity protect against the age-related impairment
  - Changes in gene activity enhance the age-related deterioration
- Association of gene variants or expression levels with longevity
- Association of the gene with accelerated aging in humans
- Regulation of genes associated with aging

By ‘changing the gene activity’, we refer to any specific effect on a gene or its product that affects its transcription, translation, or the activity or stability of its product. A particular intervention method, including gene knockout, RNA interference, additional copies of a gene, therapy with a vector containing a dominant-negative version of the gene, treatment with a protein agonist and others, is indicated for each experiment.

**Figure 2.**
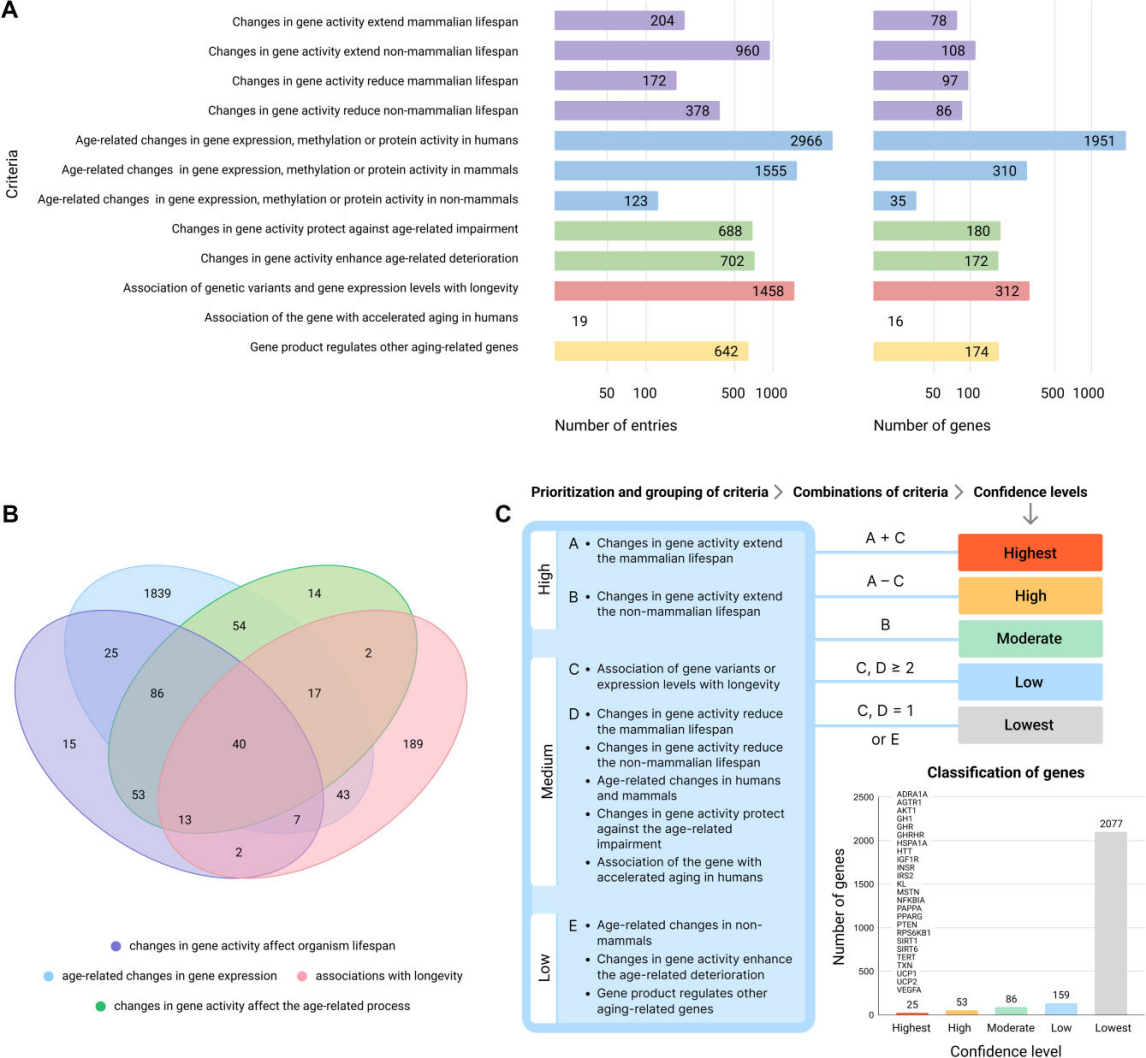
Gene selection criteria and assigning a confidence level to a gene. A — the number of entries and individual genes per criterion (the criteria were matched with the gene only if the experiment result was significant, while the number of entries included non-significant results); B — intersections of genes satisfying the criteria corresponding to four of the six study types; C — A + C — a gene meets two selection criteria, A and C, at the same time; A – C — a gene meets criterion A but doesn’t meet criterion C; B — a gene meets criterion B; C, D ≥ 2 — a gene meets two or more criteria from lists D and/or C; C, D = 1 or E — gene meets only one criterion from list D or C or any number of criteria from list E.

The gene was added to the Open Genes database if it met at least one of the 12 criteria. Figure 2 shows the Open Genes database statistics. Open Genes currently contains 2402 genes associated with aging.

Filters on the main genes table (https://open-genes.com/genes) of the database allow users to generate lists of genes according to selection criteria, combination of criteria and many other parameters.

#### Ranking the criteria

Obviously, meeting a single of these 12 criteria is not sufficient enough to establish a link between genes and human longevity. Therefore, we assigned the criteria a high, medium, or low priority (Figure 2).

It is unclear whether changing the activity of genes that decrease lifespan in the opposite direction will lead to an increase in life expectancy. We believe that genetic manipulations that increase lifespan, provide stronger evidence for considering genes as an aging therapy target than manipulations that decrease lifespan. Depending on the results, lifespan experiments were assigned different levels of priority. A high priority was placed on evidence related to the increase in the lifespan of model organisms.

Medium priority was given to data on protection against age-related impairment, the association of a gene with accelerated aging in humans, the decrease in the lifespan of model organisms, age-related changes in gene expression in humans and mammals, and associations of gene variants with longevity. All other criteria, such as deterioration in age-related processes, regulation of other age-related genes and age-related changes in non-mammals, were assigned a low priority.

#### Division of aging-associated genes to different confidence levels

We divided all genes into five confidence levels: highest, high, moderate, low and lowest (Figure 2). The group of genes with the highest confidence level includes 25 human genes that increase the lifespan of mammals and are known to have variants associated with human longevity.

We proceeded under the assumption that it remains unclear if we can extrapolate the results from model organisms to humans. At the same time, population studies of the genetics of longevity often show conflicting results and only can suggest the influence of a particular gene on the predisposition to longevity. Therefore, we used data on the association with longevity as an additional parameter for genes that extended the mammalian lifespan. Table 1 shows Open Genes data on these 25 genes. We suggest that some of these genes might be potential targets for human aging therapy.

**Table 1.** Genes associated with human longevity and increased mammalian lifespan

Gene symbol	Gene name	Organism, ortholog and number of studies/entries* for data on increased lifespan	Effect on gene function, increased lifespan	Longevity polymorphisms
ADRA1A	Adrenoceptor alpha 1A	*Mus musculus*, Adra1a, 1/1	Gain of function	8:26614201
AGTR1	Angiotensin II receptor type 1	*Mus musculus*, Agtr1a, 1/1	Loss of function	rs422858, rs275653, rs2307085
AKT1	AKT serine/threonine kinase 1	*Mus musculus*, Akt1, 1/2	Loss of function	rs3803304
GH1	Growth hormone 1	*Rattus norvegicus*, Gh1, 2/3	Loss of function	rs2665802
GHR	Growth hormone receptor	*Mus musculus*, Ghr, 5/12	Loss of function	rs4130113, rs2940923, rs2940935, rs3764451, rs12153009, rs6883523, rs4866941, rs4292454, rs6887528, GHR-Indel
GHRHR	Growth hormone releasing hormone receptor	*Mus musculus*, Ghrhr, 1/1	Loss of function	rs2267723, rs4988505, rs2228078
HSPA1A	Heat shock 70kDa protein 1A	*Mus musculus*, Hspa1a, 1/2	Gain of function	rs1008438
HTT	Huntingtin	*Mus musculus*, Htt, 1/1	Gain of function	rs61348208
IGF1R	Insulin-like growth factor 1 receptor	*Mus musculus*, Igf1r, 1/2	Loss of function	rs12437963, rs2272037 rs2229765
INSR	Insulin-like growth factor 1 receptor	*Mus musculus*, Insr, 1/1	Loss of function	rs2252673
IRS2	Insulin receptor substrate 2	*Mus musculus*, Irs2, 2/5	Loss of function	Gly/Asp
KL	Klotho	*Mus musculus*, Kl, 1/4	Gain of function	rs1207362, rs9536314, rs2283368, rs9527026
MSTN	Myostatin	*Mus musculus*, Mstn, 1/1	Loss of function	rs1805086
NFKBIA	NFKB inhibitor alpha	*Mus musculus*, Nfkbia, 1/1	Gain of function	rs2233407, 14:35875417
PAPPA	Pappalysin 1	*Mus musculus*, Pappa, 3/4	Loss of function	rs449807, rs4837525
PPARG	Peroxisome proliferator activated receptor gamma	*Mus musculus*, Pparg, 2/2	Gain of function	rs1801282, rs1175542
PTEN	Phosphatase and tensin homolog	*Mus musculus*, Pten, 1/2	Gain of function	rs1022427
RPS6KB1	Ribosomal protein S6 kinase B1	*Mus musculus*, Rps6kb1, 1/2	Loss of function	rs1051424, rs201316437
SIRT1	Sirtuin 1	*Mus musculus*, Sirt1, 1/3	Gain of function	rs7896005, rs4746720, rs4746720
SIRT6	Sirtuin 6	*Mus musculus*, Sirt6, 1/4	Gain of function	rs107251
TERT	Telomerase reverse transcriptase	*Mus musculus*, Tert, 2/3	Gain of function	rs4975605, rs2736100, rs2853690, MNS16A, rs2853676, rs2853677
TXN	Thioredoxin	*Mus musculus*, Txn1, 2/4	Gain of function	rs3808888
UCP1	Uncoupling protein 1 (mitochondrial, proton carrier)	*Mus musculus*, Ucp1, 2/8	Gain of function	haplotype: rs1800592 + C-3740A
UCP2	Uncoupling protein 2 (mitochondrial, proton carrier)	*Mus musculus*, Ucp2, 1/2	Gain of function	rs660339, rs659366, rs7109266, Ala/Val
VEGFA	Vascular endothelial growth factor A	*Mus musculus*, Vegfa, 1/2	Gain of function	rs699947, rs13207351, haplotype: rs699947 + rs13207351 + rs1570360 + rs2010963

* Entries—individual experiments with similar or different line, sex, conditions, genotypes.

The groups with high confidence level include all genes that increased mammalian lifespan excluding genes that at the same time were associated with human longevity. The moderate group includes genes that have extended the lifespan of non-mammals. Genes that have extended life only in non-mammals represent a wide field for research on their effect on mammalian lifespan.

Only those genes that met at least two of the six medium-priority criteria were included in the low-confidence group. It is unclear whether these genes affect lifespan, but there is indirect evidence for their association with aging; consequently, they might be interesting as novel targets for further lifespan experiments. Genes meeting only one of the criteria with medium priority and/or criteria with low priority were included in the last group with the lowest confidence. Thus, we received a list of 323 human genes that meet the criteria with high priority or at least two different criteria with medium priority (Figure 2). We added a filter that allows sorting genes according to the confidence level of their association with human aging (https://open-genes.com/).

Our classification has limitations. For instance, genes FOXO3 and APOE, whose association with human longevity has been confirmed in numerous population studies, are not in the ‘highest’ and ‘high’ groups due to the lack of experimental evidence demonstrating their ability to increase mammalian lifespan. Downregulation of another well-known gene, mTOR, increased the lifespan of all studied model organisms (worms, flies and mice), but no statistically significant associations of this gene with human longevity were found. Of course, this does not rule out the possibility that mTOR is associated with human longevity, and this gene was assigned to the group of genes with a high confidence level. Thus, genes with the highest confidence level are a special group of genes that simultaneously meet two parameters. They extended the lifespan of animals and have been shown to be associated with human longevity.

However, we collected numerous experimental parameters and provided various filters for the genes; therefore, users can apply their own analytical methods and scenarios for selecting aging-related genes.

### Open Genes data

#### Impact of gene activity changes on the lifespan and age-related processes

When collecting data on the effects of genetic manipulations on model organisms’ lifespans, we accounted for the fact that experiment outcomes are dependent on the experimental procedure. Depending on study design and data availability, up to 35 parameters for each manipulation are specified, including absolute values of lifespans of control and experimental groups, tissue specificity of an intervention, size of control and experimental groups, and the animals’ maintaining conditions (temperature, diet, number of animals in a container, cage, or plate). All the parameters of the experiments are described on our website in the section ‘Open Genes data description’ (https://open-genes.com/about/articles/open-genes-data-description). A combination of these parameters permits a more comprehensive interpretation of results.

The lifespans of the same line of an organism can vary between different laboratories. For instance, according to our data, the median lifespan of control C57BL/6 mice varies from 500 plus days to >900 days in mixed-sex samples (7,8). In some cases, the control has initially shorter lifespan and an increase in lifespan through genetic manipulation does not overcome the normal life expectancy. Therefore, it is important to demonstrate the absolute values of control and experimental animals' lifespans apart from percentage changes. In addition, as will be discussed below, many interventions lead to inconsistent results in different experiments. A detailed and structured description of all experimental conditions in the Open Genes database enables comparison of lifespan experiments and a more accurate interpretation of the results.

The Open Genes database already contains 1975 entries for lifespan experiments related to the 247 human genes that are orthologs of model organisms’ genes, and 78 and 108 human genes are orthologs of genes that increase mammalian and non-mammalian lifespan, respectively. The structured data from the lifespan experiments are available on the Open Genes download page (https://open-genes.com/download).

In addition to providing detailed data on the extension and reduction of life expectancy we also provide an additional outcome of intervention: the list of processes that were improved or impaired after genetic manipulation. We also collected data from experiments in which manipulation of the gene affected the age-dependent process, but data on changes in lifespan were not obtained. During the data collection process, we have compiled a list of 50 processes, organs, functions, or systems that have been cited in the literature as indicators of aging or are affected as a result of lifespan-changing interventions was compiled. Currently, the database contains 1390 entries (273 genes) on the effect of gene modification on a particular aging-related alteration. This allows for the observation of possible mechanisms of genetic manipulation's effect on the lifespan and widens the perspective in the search for target combinations. The data are available for download at https://open-genes.com/download.

#### Aging-associated changes in gene expression, methylation, and protein activity

Numerous studies have demonstrated that aging is connected to transcriptomic (9–11) and proteomic (12) changes, modifications of gene methylation (13,14), and alterations in the ratio of protein isoforms (15–17) and their intracellular localization (18,19).

Currently, we have 4644 records on age-related alterations and 2116 genes that were shown to change expression, protein activity, or methylation level with age in 29 organisms and 224 tissues. Of these, 2965 notes and 1951 genes are human, as we focused primarily on obtaining human data.

We focused on establishing a unified data structure so that it could be universally utilized for all research purposes. For this, 13 parameters were assigned, which are essential for evaluating the quality and results of an experiment (https://open-genes.com/about/articles/open-genes-data-description).

When possible, separate results for each sex were shown along with the precise ages of the compared groups to estimate the ages at which changes most likely occurred. We present results without recalculation but specify the numerical characteristics and methods for data collection and statistical analysis.

Open Genes can be used for scanning genes, for instance, to determine whether the effect of these genes on lifespan has been assessed in model organisms or not yet, whether these genes are associated with some aging symptoms, and whether they are associated with longevity (more detailed in Open Genes instruments). The data are available on the Open Genes download page (https://open-genes.com/download).

#### Association of genetic variants and gene expression levels with longevity

As with many other phenotypic features, the predisposition to longevity is a partially inherited multifactorial trait; thus, one of the factors increasing the individual's longevity chances must be a certain combination of alleles (20). We collected 1458 records on 362 genes, whose association with longevity was studied; at least 312 genes were shown to be significantly associated with longevity or human lifespan. We organized the experiment data into the following structures: sample data, study design, and study results. The data were structured using 17 experimental parameters (https://open-genes.com/about/articles/open-genes-data-description).

In addition to showing data on longevity association, we also show the available information on whether the given allelic variant is associated with increased or decreased gene expression or protein activity. For example, the rs1801195 polymorphism in the WRN locus that is associated with increased gene expression may increase the probability of longevity (21). The finding that WRN gene expression reduces and methylation increases with age in human blood mononuclear cells (22) is presented on the page for the WRN gene in our database and complements data from the previous study. Data on the effect of longevity polymorphisms on gene expression for genes that have extended life in mammals have been discussed above and are presented in Table 1. Population study data structured and collected in Open Genes can be useful for performing meta-analyses and determining of the gene therapy targets. The structured data from population studies of longevity are available on the Open Genes website (https://open-genes.com/download).

#### Origin and evolution of genes

Information about the origin and evolution of genes is essential for understanding the evolution of aging hallmarks, which was described in the recent publication (23). We utilized the method described by Capra *et al.* in their article ‘How old is my gene?’ to estimate the approximate age of the gene and gene family (24). It is difficult to determine the age of a particular gene, as genes are constantly evolving. Genes do not have precise ages, especially genes with a complex evolutionary history. The gene age is usually associated with an important event in its evolution, such as duplication, horizontal transfer, or *de novo* origin (24).

The gene is essential for studying the genetics of aging in the context of related processes or biological mechanisms. Therefore, we considered the age of the gene family based on the most evolutionarily ancient taxon that is known to possess a homologous gene with a similar function. Currently, Open Genes has 455 genes with manually-collected data on their evolution. Most gene families have been discovered in metazoans (120), prokaryotes (102) and eukaryotes (101). We manually determined the origin of the gene family by analyzing studies on molecular evolution. If such information was available, we also indicated the origin of each gene. On the gene page, there is a comment on the evolutionary age of the gene and gene family, which provides a brief rationale with literature references, as well as a description of the main events in gene evolution. In addition to the manually collected data, sequence conservation data were parsed from HomoloGene NCBI (6). The data are available at https://open-genes.com/download.

Consideration of the evolution of genes and molecular pathways might be useful for discovering the potential therapeutic targets among aging-associated genes in animal models.

#### Association of genes with hallmarks of aging

López-Otín *et al.* distinguished nine hallmarks of aging, or mechanisms that mutually participate in human aging (25). Based on these nine ‘synthetic’ hallmarks, Maël Lemoine identified 20 ‘analytical’ hallmarks of aging (23). In a recent publication, López-Otín *et al.* described the nine hallmarks of aging in greater detail and added three new ones (26). Each hallmark represents a vital biological process that involves multiple associated genes and is disrupted during aging.

We chose to establish a connection between the genes in our database and biological processes, whose dysregulations characterize human aging. We used the Gene Ontology tree of biological processes and molecular functions to link each gene to a particular hallmark of aging. Each hallmark of aging was manually matched with the corresponding nodes of the GO process and function trees (26,27). Currently, each new gene in Open Genes is automatically assigned to a hallmark or multiple hallmarks of aging based on the GO terms associated with that gene. For example, the TERT gene is associated with the ‘telomere attrition’ aging hallmark since it participates in the GO biological processes ‘telomere maintenance’ and ‘telomere maintenance via telomerase’. Genes can be filtered by hallmarks of aging on the Open Genes homepage https://open-genes.com/.

Thus, putative associations between a gene and a hallmark of aging are shown based on the gene's involvement in the corresponding biological process. We considered 20 aging hallmarks offered by Lemoine (23), added ‘disabled macroautophagy’ from the last review by López-Otín *et al.* (28), and completed this list with two additional hallmarks of aging suggested in the recent publication of Pun *et al.* (29) A complete list of hallmarks of aging and their corresponding nodes on the GO tree is presented in Supplementary Table S1.

#### Filters and searching

During the development of Open Genes, we aimed to simplify the search for any data pertaining to the genetics of aging. The main table of the database provides the following information for each gene:

- HUGO symbol and gene name
- Criteria for adding a gene to the database
- Confidence levels of the genes
- Number of entries in each study type
- Association with hallmarks of aging
- Association with diseases and disease categories according to ICD-10
- Protein classification according to the Human Protein Atlas
- The origin of the gene and the gene family
- Conservation according to HomoloGene NCBI

**Figure 3.**
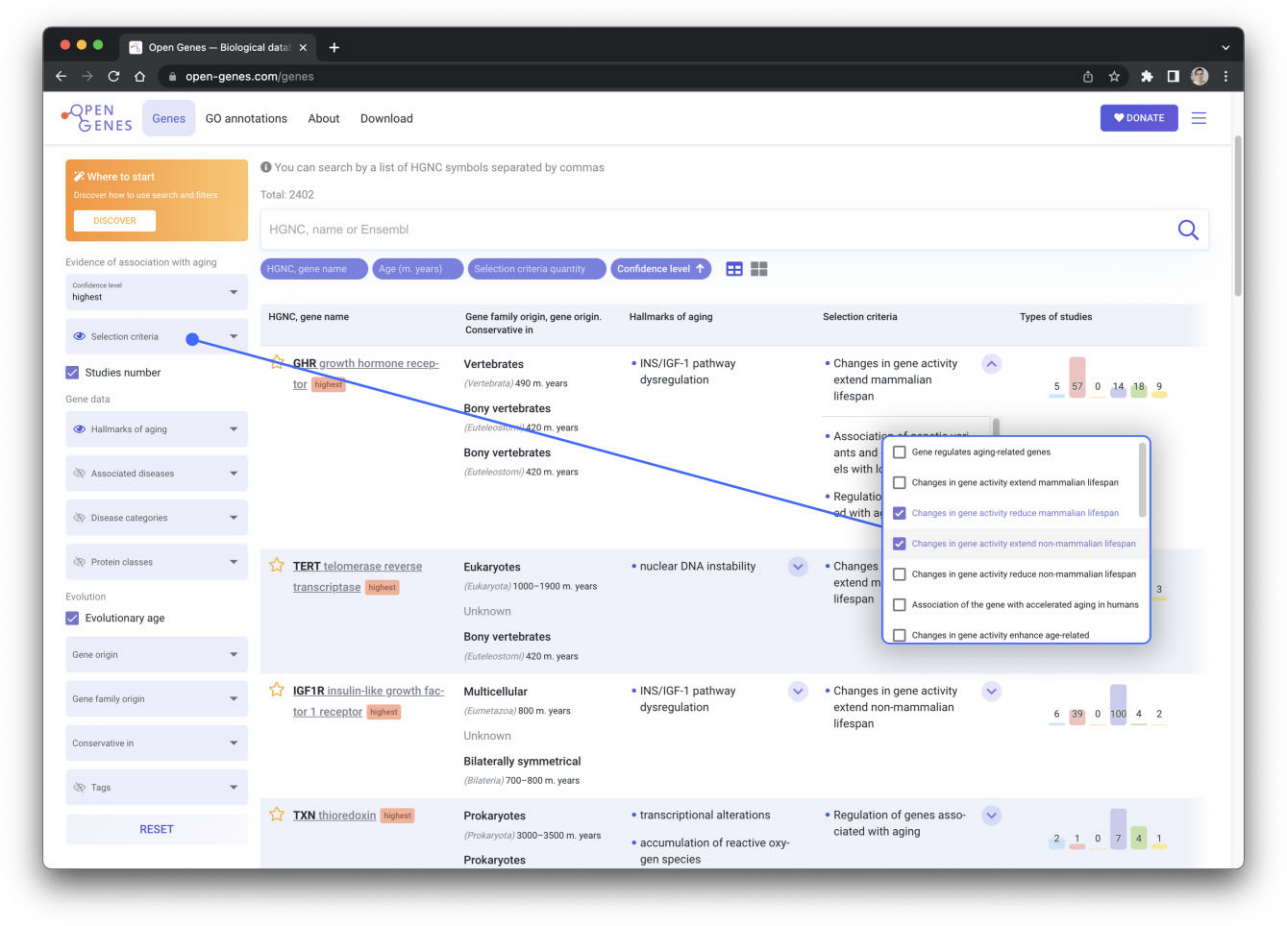
Searching and filtering in the Open Genes database.

**Figure 4.**
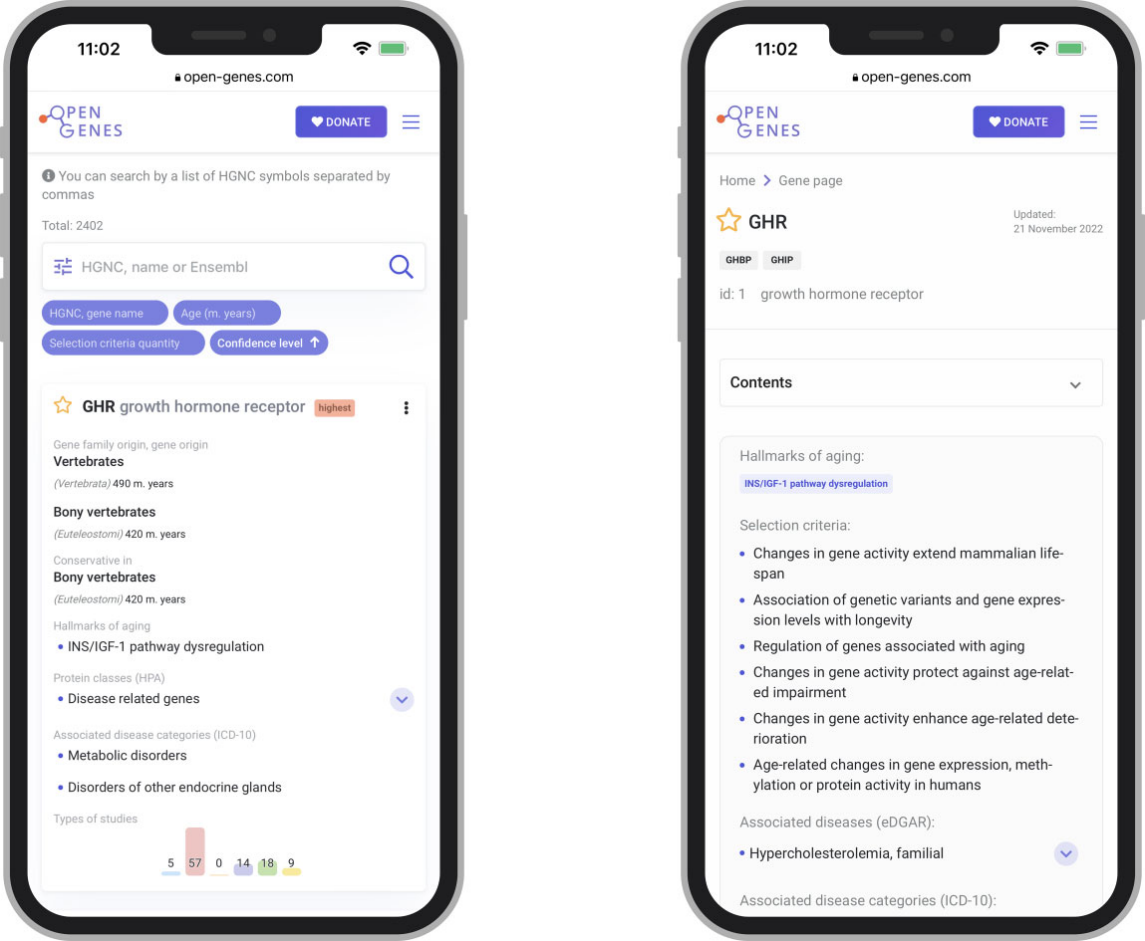
Mobile version of the Open Genes database.

The genes in the table are filtered by each parameter individually and by a combination of parameters (Figure 3). We have also developed a convenient mobile version of the database (Figure 4). The search by HGNC, name and GO terms is available in Open Genes. The website also provides an option to search by the gene list, allowing users to determine which of the genes on their list are included in Open Genes and what is known about their connection to aging.

The user can download separate tables for each type of study from https://open-genes.com/download. The page for each gene contains all the information, both manually collected and added from other databases, including tables with all experiments’ details and a description of the gene's evolution.

### Data from other databases

Open Genes imports a significant amount of information about genes from other databases in an effort to maintain all essential genetic data in the most accessible format. General data for the genes are automatically obtained monthly by parsing from third-party database APIs (https://open-genes.com/about/articles/reference-list). We import the gene name, its synonyms, transcript information, as well as gene description, orthologs in vertebrates and conservation from the NCBI (30), NCBI HomoloGene (https://www.ncbi.nlm.nih.gov/homologene) and MyGen.info (31) (https://mygene.info) databases. Gene location on chromosomes is obtaining from HUGO database (32). Orthologs for Caenorhabditis elegans and Drosophila melanogaster are extracted from WormBase and FlyBase databases, respectfully (33,34). In addition, the protein description is included from UniProt (35). Some information is retrieved from the Human Protein Atlas, including tissue-specific data on gene expression levels, protein classes, cell-type and tissue-type specificity, and the predicted intracellular and extracellular localization of the protein (36). We use the GO database to retrieve information regarding biological processes, molecular functions and cellular components (26,27). To link a gene with different hallmarks of aging, we use the GO terms tree provided by QuickGO REST APIs (https://www.ebi.ac.uk/QuickGO/) (37). Data about disease associations are obtained from the eDGAR database (38). We obtain ICD codes for diseases associated with genes and the ICD diseases tree from the Orphanet database (39) (http://www.orpha.net) and ICD-11 (40) (https://icd.who.int/browse11).

### Analyzing Open Genes data

Using the Open Genes data, we analyzed the consistency between lifespan experiments performed on mammals and population studies of human longevity. We also classified the genes according to the degree of inconsistency in the results of lifespan experiments on different animal models. By using similar scenarios to analyze the experimental data that we have collected, users can obtain lists of genes of interest that correspond to certain parameters.

#### Effect of gene activity on mammalian lifespan and longevity in humans

**Figure 5.**
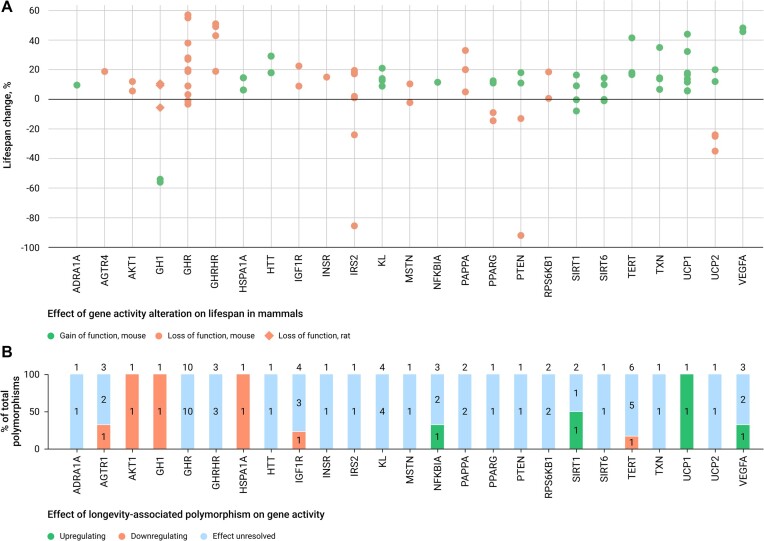
Effects of gene activity on mammalian lifespan and longevity in humans. (**A**) Change in the mean/median lifespan of mammals (depending on available data) caused by genetic intervention; each dot is an individual experiment; (**B**) the amount of longevity-associated polymorphisms and their effect on gene activity; the numbers above and within the bars represent the number of gene polymorphisms associated with longevity.

For 25 genes with a high confidence level (Table 1), we analyzed the effect of polymorphism associated with human longevity on gene expression, we collect such data if it is available. The results of this analysis are presented in Figure 5.

For 10 of the ‘highest confidence level’ genes, there are data on the effect of longevity-associated polymorphisms on the level of gene expression or protein activity. Interestingly, in eight instances, these data agree with the results of experiments on mammalian model organisms. Centenarians are carriers of variants of genes associated with increased gene expression, and upregulation of these genes extends the lifespan of animals (NFKBIA, SIRT1, UCP1 and VEGFA). Conversely, genes that must be suppressed for animal longevity are downregulated in centenarians (AGTR1, AKT1, GH1 and IGF1R).

Thus, using a variety of parameters specified for each gene and experiment in the database, users can narrow down groups of genes to a few genes and isolate special genes of interest.

#### Consistency of the lifespan experiments results

The effect of gene activity modification on the lifespan of a model object is the most direct and obvious evidence of the gene association with life expectancy. Using Open Genes data we classified genes into six groups based on the effect of loss (LF) or gain (GF) of gene function on the lifespan of model organisms. By ‘loss of gene function’, we refer to the interventions that decrease gene expression or gene product activity, such as gene knockout or knockdown, RNA interference or therapy with a specific gene product inhibitor. ‘Gain of function’ includes overexpression of a gene with additional copies of the gene or therapy with a vector expressing the given gene, a mutation that increases the stability or activity of the gene product, and therapy with specific inducers of the gene product.

The first group includes genes with consistent data on both LF and GF: suppressing the gene activity always extends the lifespan, whereas overexpressing the gene leads to decreased life expectancy, and vice versa. Activation and repression of these genes are well studied, and the effect of an intervention is predictable.

**Figure 6.**
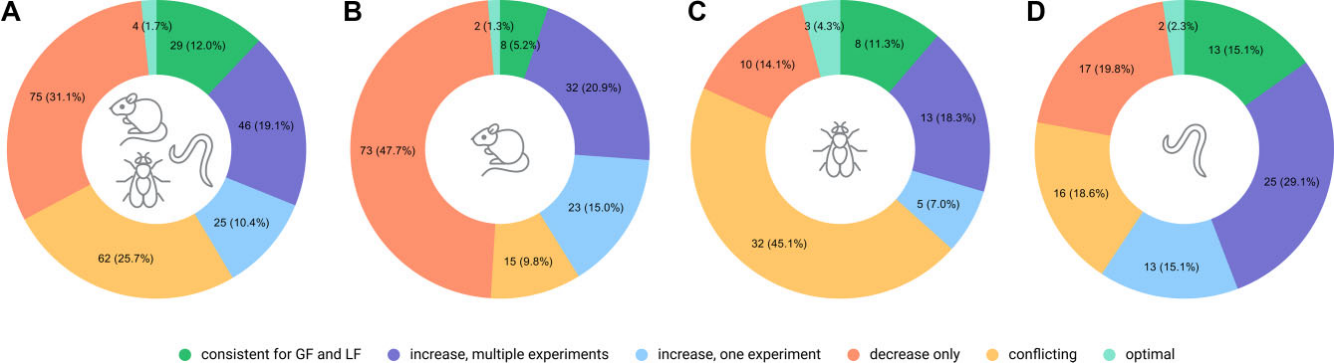
Genes groups based on the consistency of data from lifespan experiments. (**A**) all organisms (241 human homologs); (**B**) mammals (mice, rats, hamsters, rabbits, 153 human homologs); (**C**) *Drosophila melanogaster* (71 human homologs); (**D**) *Caenorhabditis elegans* (86 human homologs).

The second and third groups include genes for which there are consistent results from several experiments or a single experiment, respectively, indicating that gene activity modulation increases life expectancy; however, the reversal of gene activity has not been studied. The fourth group includes genes with conflicting experimental data; for example, in some cases, LF or GF increased lifespan, while in others it decreased. The fifth group includes genes whose activity modification leads to a decrease in the lifespan of model organisms as a result of accelerated aging. Finally, genes were included in the sixth group if any change in their activity, either a loss or gain of function, reduced the lifespan of model organisms. It can be assumed that any modification of such genes is not beneficial since their activity is already optimal for longevity; therefore, we called this group of genes ‘optimal’. We performed this analysis for a combined group comprised of all organisms, as well as separately for mammals, flies, and worms. The results of the analysis are represented in Figure 6.

The least controversial results were obtained on mammals (9.8%), and the most controversial results were obtained on flies (45.1%). It is important to note that some of the conflicting results might be explained by differences in experimental conditions, for example, obtained genotypes, sexes or lines of organisms, tissue specificity, etc. Data on lifespan experiments require a deeper analysis to reveal true contradictions. Only eight genes (5%) have consistent results in both gain and loss of function experiments on mammals (Table 2).

**Table 2.** Genes with the most consistent and comprehensive data on mammalian lifespan experiments

Human gene	Lifespan effect in mammals
CISD2	GF increases, LF decreases
KL	GF increases, LF decreases
PAWR	GF increases, LF decreases
PPARG	GF increases, LF decreases
PTEN	GF increases, LF decreases
SIRT6	GF increases, LF decreases
UCP2	GF increases, LF decreases
IKBKB	LF increases, GF decreases

GF, gain of function; LF, loss of function.

Five of the genes with the most consistent and full data on mammalian lifespan experiments, KL, PPARG, PTEN, SIRT6 and UCP2, were also shown to be associated with human longevity and were included in the ‘highest confidence’ group (Table 1). All human genes classified into six groups based on the consistency of the lifespan experiment results are presented in Supplementary Table S2.

The ratio of consistent and conflicting results allows us to determine which model organisms are more suitable for lifespan experiments. Most of the conflicting results were obtained in experiments with flies. This may indicate that the lifespan of flies is highly dependent on various factors, many of which can be difficult to control. A deeper analysis of contradictions, taking into account such parameters as genotype, sex, temperature, tissue specificity and others, can be useful to determine what factors affect the results of the experiment.

## Discussion

We have collected data on 2402 genes and obtained a list of 323 genes most likely associated with aging, classifying the genes by confidence levels. We have provided detailed and structured data for each experiment, which can be downloaded from the database website. We have added associations with diseases and hallmarks of aging. In addition, we have developed convenient filters for sorting genes on the website by a variety of parameters.

Currently, we are developing an algorithm for ranking each experiment based on study type, outcome, significance, sample size and other factors that might be used to evaluate the quality of the experiment and the impact of the experiment on the confidence of the genetic target for aging therapy. This will allow us to rank all genes by the number, quality, and consistency of experiments confirming their association with human aging. Thus, by applying these analysis instruments to the updated and augmented data, we will be able to precisely determine which targets can be most reliably used for aging therapy in accordance with the latest data, which targets require further study, and which experiments precisely must be conducted to confirm genetic targets with a low confidence level.

The completeness of the Open Genes database is still limited, especially with regard to the experimental data on non-mammalian model organisms as we primarily focused on data from mammalian studies at this stage. Data from studies on worm and fly orthologs of human genes were collected predominantly for 307 human aging-associated genes from the GeneAge database list (5). Numerous studies have been conducted on other worm and fly orthologs of human genes, and we are currently incorporating data from these studies into the database. We also began collecting data on combinations of genetic interventions.

While working on the Open Genes database, we developed the CMS for internal use. We created forms for each type of study with separate fields to be filled, covering all aspects of the study and maintaining the standardized format of entered data. We intend to improve the CMS so that users can submit data from published studies by uploading CSV documents or manually filling out forms. In addition, we would like to supplement data validation with a version control system (VCS): data proposed by a user will be verified by curators before being displayed on our website.

Finally, we plan to use the collected and structured data to train neural networks. Manual collection of many experimental parameters takes a vast amount of time. The relevance of the database can be maintained by collecting data using trained neural networks with further verification by moderators.

## Materials and methods

### Study selection

The first step was to search for studies related to each of the 307 genes offered by the GeneAge (6) database using a query that consisted of a gene symbol combined with the following keywords in PubMed: ‘aging’, ‘longevity’, ‘gene knockout aging’, ‘gene overexpression aging’, ‘gene modification aging’, ‘expression aging’, ‘mRNA level aging’, ‘protein level aging’, ‘expression young old’, ‘polymorphism longevity’, ‘polymorphism aging’, ‘gene variant longevity’, ‘age-related’, ‘age-associated’, ‘lifespan’, ‘senescence’. At the second stage, we searched for publications related to the types of studies of interest using the following keywords: ‘gene knockout aging’, ‘gene overexpression aging’, ‘gene modification aging’, ‘expression aging’, ‘mRNA level aging’, ‘protein level aging’, ‘expression young old’, ‘polymorphism longevity’, ‘polymorphism aging’, ‘gene variant longevity’. The studies were then manually reviewed for eligibility by a moderator.

For the lifespan studies, the same selection criteria that were used for the GeneAge database were taken into consideration. We excluded studies in wich the mutation was lethal at an early age, causing rather severe health complications than accelerated aging (6). Studies representing changes in the onset or severity of health impairments were included only when they met the following criteria: (a) the studied process was age-related, as reported in the literature; (b) the study showed that the age-related impairment manifested earlier or later and was stronger or weaker in aged experimental animals than in aged control animals. Animal models of diseases were excluded.

For the studies on age-related expression changes, gene methylation, or protein activity, we applied the following criteria: (a) the study used *in vivo* models; (b) changes must be associated with age, not the disease. Both significant and non-significant results from the studies on polymorphisms and longevity associations were included, as well as data on associations between gene product levels and longevity. For choosing the studies that specifically confirm the association of mRNA or protein levels with longevity and not just age-related changes, the following criteria were used: (a) the heritability of association of the expression level with longevity was confirmed in a cohort of centenarians’ descendants; (b) expression levels were evaluated before reaching the longevity age; thus, gene expression levels can be used to predict longevity.

### Platform and software

All code is available on GitHub (https://github.org/open-genes) under the Mozilla Public License 2.0. Open Genes consists of several applications. They could be generally divided into two groups: backend (server applications) and frontend (client applications). The backend infrastructure consists of a database, an API, console scripts for data parsing and updating, and a content management system (CMS) for biologists. The frontend is presented with a single-page application—the Open Genes website. Both types of applications also have scripts and configuration files for integration, deployment and testing.

### Frontend

The frontend is being developed using a modern technology stack. We adhere to the principles that dictate isolating dependencies and encapsulating application logic in separate modules at different levels. It offers scalability and facilitates old code maintenance.

The Open Genes website is a data-driven single-page application developed on Angular 13 and written in TypeScript. The web application allows users to browse, search, filter, sort, group and share data from the Open Genes API using a graphical interface. The website is responsive and supports mobile devices.

### Api

API (Application Programming Interface) provides programmatic access to all collected data in JSON format. We adhere to a twelve-factor methodology and use principles of layered architecture, which allow us to isolate the logic from the framework and infrastructure.

Open Genes API is a REST API written in Python 3 and based on the FastAPI framework. API endpoints give access to data in the database on genes, research data, and entities associated with genes, such as diseases, age-related processes, orthologs, Gene Ontology (GO) terms, protein categories, aging mechanisms, gene origin, and other categories.

### Console scripts

Backend applications use console scripts for various purposes, primarily for obtaining data from numerous sources and updating the database.

### Database

The data are stored in a relational database management system, MySQL 8, in nearly 80 tables. The database structure is normalized o ensure that all the data on genes and experiments are consistent with the relevant relations between them.

## Supplementary Material

gkad712_Supplemental_File

## Data Availability

Open Genes has several options for retrieving data. The whole database can be downloaded as a MySQL dump file from our website on a ‘Download data’ page. Users can download separate datasets in TSV format (https://open-genes.com/download). REST API best fits for programmatic access to the database. Open Genes API provides GET endpoints for retrieving data for genes and structured research descriptions along with many various entities described in this article. It also provides many parameters for filtering and sorting. Full interactive API documentation in Swagger (OpenAPI) format can be found here: https://open-genes.org/api/docs (permanent DOI: https://doi.org/10.5281/zenodo.8475).

## Code availability

All code is available on GitHub (https://github.org/open-genes) under Mozilla Public License 2.0 (permanent DOI: https://doi.org/10.5281/zenodo.8475).
